# Improved mental health for women receiving infant mental health home visiting: a randomized controlled trial

**DOI:** 10.3389/fpsyg.2025.1597655

**Published:** 2025-10-09

**Authors:** Jennifer M. Jester, Ann M. Stacks, Jessica L. Riggs, Holly E. Brophy-Herb, Maria Muzik, Katherine Rosenblum, Michigan Collaborative for Infant Mental Health Research, Emily Alfafara, Carla Barron, Holly E. Brophy-Herb, Nora L. Erickson, Hiram E. Fitzgerald, Alissa C. Huth-Bocks, Meriam Issa, Jennifer M. Jester, Megan M. Julian, Jamie M. Lawler, Rena Menke, Alyssa S. Meuwissen, Alison L. Miller, Maria Muzik, Larissa N. Niec, Jerrica Pitzen, Julie Ribaudo, Jessica Riggs, Katherine L. Rosenblum, Sarah E. Shea, Paul Spicer, Ann M. Stacks, Chioma Torres, Laurie Van Egeren, Rachel Waddell, Christopher L. Watson, Deborah J. Weatherston, Kristyn Wong

**Affiliations:** ^1^Michigan Medicine, Department of Psychiatry, University of Michigan, Ann Arbor, MI, United States; ^2^Merrill Palmer Skillman Institute, Wayne State University, Detroit, MI, United States; ^3^Department of Human Development and Family Studies, Michigan State University, East Lansing, MI, United States

**Keywords:** infant mental health, postpartum anxiety, postpartum depression, post-traumatic stress disorder, home visiting

## Abstract

**Introduction:**

This study evaluates the impact of a home-based infant mental health intervention on maternal mental health symptoms. Prevalence rates of maternal depression, anxiety and trauma symptoms are quite high during the postpartum period and can contribute to ruptures in the parent–child relationship and infant development. While some infant mental health interventions improve depression, less is known about the impact of home-based or attachment-based psychotherapeutic interventions on maternal anxiety or post traumatic stress disorder.

**Method:**

Using a randomized controlled trial design, mothers with infants were recruited and randomized to infant mental health home visiting (IMH-HV; *n* = 38) or a control group (*n* = 35). However, five dyads who were assigned to the treatment group but received no treatment were omitted from the analysis, for a per-protocol analysis of 68 mothers. Mothers reported on their depression (Patient Health Questionnaire, PHQ-9), anxiety (General Anxiety Disorder, GAD-7), and trauma symptoms (PTSD Checklist, PCL-5) at baseline, and six and 12 months later.

**Results:**

The mothers in the per-protocol treatment group (*n* = 33) demonstrated greater decreases in mental health symptoms over the 12 months of the study than those in the control group (*n* = 35) (slope effects: for depression (−0.19, *p* = 0.015), anxiety (−0.13, *p* = 0.058), and trauma (−0.46, *p* = 0.057)).

**Discussion:**

Results suggest that IMH-HV services are effective in reducing mental health symptoms for mothers who actually received treatment.

**Clinical trial registration:**

NCT03175796

## Introduction

Infant Mental Health Home Visiting (IMH-HV) is an attachment-based, psychotherapeutic intervention, delivered by trained mental health professionals to support the social, emotional, and relational development of infants and toddlers. Ongoing parental stress and material hardship, a history of relational trauma, and current mental health challenges can contribute to ruptures in the parent–child relationship (Feldman et al., 2009; Slomian et al., 2019) and have a negative effect on social–emotional and cognitive development (Riggs et al., 2022; Toth et al., 2006). In Michigan, IMH-HV programs are offered within each county’s Community Mental Health System, through Medicaid and other federal grant mechanisms. Families are eligible for IMH-HV if they are pregnant or have a child up to age three, and are experiencing any of the following risk factors: difficulty with bonding or responsive caregiving, environmental or economic stressors, parent mental health concerns, a history of relational trauma or loss, involvement in the child welfare system, or child developmental concerns. The Michigan Infant Mental Health Research Collaborative partners with these programs to evaluate IMH-HV and develop its evidence base, which is required for Medicaid reimbursement. An evaluation of the Michigan Model of IMH-HV in a community-based open trial and a randomized controlled trial (RCT) showed promising results when evaluating the impact on the parent–child relationship, parenting, and child outcomes. In the open trial, IMH-HV was associated with decreased harsh parenting and decreased risk for child maltreatment (Julian et al., 2021; Julian et al., 2023), increased maternal sensitivity (Rosenblum et al., 2020), improved parental reflective functioning (Stacks et al., 2019, 2022), and improved child development (Stacks et al., 2020). In the RCT we found that mothers with low to moderate PTSD symptoms receiving IMH-HV reported significantly improved child social–emotional well-being as compared to mothers in the control condition (Ribaudo et al., 2022). Additional work on examining child outcomes in the RCT is underway. However, there remains a need to assess the impact of the Michigan Model of IMH-HV on maternal mental health symptoms, which is the focus of this study.

Prevalence rates of maternal depression, anxiety, and trauma symptoms are quite high; approximately 19% of women (ages 20–39) in the U. S. report symptoms consistent with depression (Brody and Hughes, 2025), and 1 in 8 women who experience depression symptoms during the postpartum period (Bauman et al., 2020). Further, the prevalence of depressive symptoms is disproportionately higher among women in low-income settings. For example, more than half of mothers in Early Head Start reported experiencing significant depressive symptoms (Chazan-Cohen et al., 2007). In addition, roughly 19% of adults in the U. S. reported an anxiety disorder in the last year, with prevalence rates higher in women than in men (Goodman et al., 2016; National Institutes of Mental Health, 2022). In the first postpartum year, up to 20% of mothers experience anxiety disorders (Goodman et al., 2016). Rates of posttraumatic stress symptoms or PTSD diagnoses are also common among mothers, with up to 18% of postpartum mothers experiencing elevated levels of PTSD symptoms (Beck et al., 2011). The majority of mothers participating in home visiting programs meet criteria for depression (Ammerman et al., 2010) and have experienced a childhood trauma or adversity (Mersky and Janczewski, 2018). Some parents experiencing mental health challenges are also often aware of how their mental health is linked with their parenting. For example, during an interview to assess reflective functioning in the open trial, one parent spontaneously described how her depression impacted her parenting, underscoring the significance of examining IMH-HV effects on maternal mental health in the current study:

*“Sometimes my depression gets the best of me, and it’s like I do not think that [child] gets the full, like me. Sometimes I just do not want to be bothered with her, or like anybody, cause it’s like I want to be in my own zone. And then, like it makes me think like …I’m not doing a good job as a parent [starts crying]. Sometimes I feel like I do not do a good job, like I complain too much, and sometimes I’m mean to her, and she do not deserve it…. I yell at her cause I’m not feeling good, but she do not deserve it, it’s not her fault.”*

Although some attachment-based interventions and home visiting programs have shown improvement in depression, much less has been published about the impact of home-based or attachment-based psychotherapeutic interventions on maternal anxiety, despite anxiety and depression being highly co-morbid (Kalin, 2020). Additionally, while home-based attachment interventions are often used in the context of child trauma symptoms, maternal PTSD symptoms are often not directly addressed via home-visiting programs, despite high prevalence (Ammerman et al., 2012). This study fills this gap by examining the impact of IMH-HV on maternal depression, anxiety, and trauma symptoms.

Home visiting programs serving parents and young children aim to build resilience, reduce risk, and foster relational well-being. They work with pregnant parents and parent-infant dyads to teach positive parenting skills, promote early learning, conduct developmental and mental health screening, and connect families to services and resources. These programs primarily serve families who face challenges to health and well-being, including poverty, low education, young parenthood, and a history of maltreatment (Maternal Child Health Bureau, 2024). Despite having similar goals, there is considerable heterogeneity in home visiting curricula; they can be delivered by volunteers, paraprofessional parent educators, community health workers, nurses, or licensed mental health professionals. In their systematic review and meta-analysis, Cibralic et al. (2025) report mixed findings across home visiting studies regarding improvements in child developmental outcomes, reductions in child abuse potential, improvements in parenting, and improvements in socioemotional and behavioral outcomes. Meta-analytic findings revealed that home visiting programs are most effective in reducing child abuse potential and improving child social and behavioral outcomes, and less consistently effective at improving parenting quality or mental health. Those that evaluate the impact of mental health tend to focus on depression.

Across most programs, home visitors report that they receive training in and are required to screen for maternal trauma and depression (Folger et al., 2022), yet they are not trained mental health professionals. Recent meta-analytic findings on home visiting programs report no significant improvement in depression symptoms (Leonard et al., 2021). It remains unclear whether psychoanalytically informed home visiting models, where mental health professionals provide services to families, are more effective. When home visiting programs employ clinically therapeutic approaches, such as cognitive behavioral therapy (CBT) to directly intervene on maternal mental health, depressive symptoms decrease (Ammerman et al., 2013; McFarlane et al., 2017; Tandon et al., 2011).

Psychoanalytically informed infant mental health interventions are grounded in attachment theory and support the social, emotional, and relational well-being of infants and young children in the context of their caregiving relationships. Infant mental health interventions work with parents and infants together, focusing on improving parental mental health, the parent–child relationship and infant outcomes. Mental health challenges can impact parent and child dyadic play behaviors (e.g., Rao et al., 2021), parenting sensitivity (Barnes and Theule, 2019; Forcada-Guex et al., 2011), and child development (Ribaudo et al., 2022; Toth et al., 2006). Moreover, maternal mental health symptoms can be problematic even at subclinical levels (Behrendt et al., 2016; Mughal et al., 2019; Skotheim et al., 2013). As such, addressing maternal mood, anxiety, and trauma symptoms is instrumental to supporting both mothers and their children. Some research suggests that individual psychotherapy and/or medication that focus on alleviating symptoms may not be sufficient to improve parenting or protect against negative child outcomes (Murray et al., 2019).

For parents who have mental health challenges, infant mental health therapists treat these symptoms by creating an emotionally safe therapeutic relationship where the parent is supported to regulate their emotions, explore unresolved grief, and reprocess trauma. Yet, there is limited evidence that these programs reduce maternal mental health symptoms, For example, Minding the Baby, an attachment-based psychotherapeutic home visiting program reported positive parenting effects but no treatment effects on maternal mental health in a community sample (Sadler et al., 2013; Slade et al., 2020). Similarly, a meta-analysis from Barlow et al. (2016) also showed that four studies that included general depression as a parent outcome showed no parent-infant psychotherapy effects on maternal mental health symptoms, despite showing effects on promoting positive parent–child relational change.

The Michigan Model for Infant Mental Health Home Visiting (IMH-HV) is conceptually similar to interventions like Minding the Baby and Parent Infant Psychotherapy. IMH-HV as a psychotherapeutic model was first developed by Fraiberg (1983) (see Weatherston, 2002; Huth-Bocks et al., 2020; Weatherston and Ribaudo, 2020). IMH-HV is a comprehensive service that attends to the psychological, emotional, relational, and concrete needs of mothers and infants and their families, expanding upon other home visiting programs that attend only to the delivery of psychotherapy, and home visiting programs that focus on building developmental knowledge and connection to community resources. This approach may provide the support needed to achieve improvements in maternal mental health not consistently evident in prior research.

IMH-HV is a manualized model (Weatherston and Tableman, 2015), where masters-level, licensed mental health providers flexibly respond to the needs of the parent and the parent-infant dyad by providing the following core intervention strategies: (1) Relationship building, (2) Providing for material needs, (3) Developmental guidance, (4) Emotional Support (5) Infant parent psychotherapy, and (6) Helping clients to develop coping skills and social support (see Table 1 for an explanation of each of these core intervention strategies). Additional details of these strategies are described in the manual (Weatherston and Tableman, 2015; Weatherston and Ribaudo, 2020). All clinicians are trained in the IMH-HV model with an Endorsement® from the Michigan Association for Infant Mental Health and receive weekly reflective supervision. Publications from the open trial describe the intervention in more detail (Weatherston et al., 2020) and explain how clinicians use various core intervention strategies throughout the course of intervention (Huth-Bocks et al., 2020).

**Table 1:** Description of core elements of the Michigan model of IMH-HV.

Relationship building	Therapists understand that the families with whom they work may have experienced relational traumas that impact their ability to trust. They work to be consistent and nonjudgemental and create a safe place for parents to explore difficult feelings and experiences. The therapist pays attention to cultural forces that underlie their own and the parents assumptions
Providing for material needs	Helping families access services to meet basic needs, including referrals for housing or food, making calls to set up medical appointments, arranging transportation, problem solving to reduce stress.
Developmental guidance	Translating the meaning of the child’s behavior for the parent through the lens of attachment and provide information about what to expect from children developmentally, especially in the context of a stressful/traumatic event; modeling a reflective stance about the meaning behind children’s behavior
Emotional support	Listen thoughtfully to parents and provide an empathic response in response to crises.
Infant parent psychotherapy	Helping parents explore their own feelings, memories, and reactions and make connections between their own early experiences, including unresolved trauma or loss, and how those experiences impact the ways in which they feel about or respond to their baby across generations and to process feelings associated with those experiences. The therapist helps the parent recall and repair relational misattunements
Helping clients to develop coping skills and social support	Parents may feel isolated and therapeutic work may involve resolving conflicts and identifying new sources of support.

## Materials and methods

This was a university-based trial. Mothers were recruited via community flyers, referrals from medical providers, or contacted from a registry of women who recently gave birth. Participants were randomly assigned to control and treatment groups, using *a priori* urn randomization to create equivalence in baseline distribution of maternal adverse childhood experiences, symptoms of depression, and family income across treatment and control groups. The treatment group was offered IMH-HV services by one of three licensed mental health clinicians trained in the intervention.

As noted above, the Michigan Model of IMH-HV services is unique in that it is a manualized intervention that blends traditional home visiting approaches (e.g., provision of developmental guidance, connection to needed resources), with traditional mental health treatment approaches to improve maternal mental health (e.g., addressing symptoms, improving social support, psychotherapy, attachment-based developmental guidance), within the context of an attachment-informed therapeutic relationship with a central goal of improving the parent-infant/toddler relationship. See below for additional information about IMH-HV as delivered in this trial.

### Participants

Eligible mothers were at least 18 years old and had legal custody of a child less than 25 months of age at the time of enrollment. As described previously, families receiving IMH-HV services in Michigan are Medicaid-eligible and have an additional risk factor related to parenting. To recruit a sample similar to those who receive IMH-HV in the community, participants needed to endorse two of the following issues: (1) probable depression diagnosis, indicated by Patient Health Questionnaire (PHQ-9) score > 9 (41%); (2) report of parenting challenges or difficulty with their child (70%); (3) parent’s retrospective report of Adverse Childhood Events from their childhood of > 2 (67%); and/or (4) eligibility for public services based on income and household size (63%). Participants were randomly assigned to treatment (*n* = 38) and control groups (*n* = 35).

### Measures

#### Maternal mental health

Mental health symptoms were measured at baseline, 3, 6, 9, and 12 months later. Anxiety symptoms were assessed using the Generalized Anxiety Disorder Questionnaire (GAD-7; Spitzer et al., 2006), a 7-item screening measure used to assess the presence and severity of symptoms commonly associated with anxiety. Participants rated how often they experienced various problems in the last 2 weeks on a 4-point Likert-type scale (0 = “Not at all” to 3 = “Every day”; total score 0–21). Internal consistency for the measure was good (*α* = 0.89–0.93).

The Patient Health Questionnaire (PHQ-9; Kroenke et al., 2001) is used to screen, measure, and monitor depression symptom presence and severity with 9 items corresponding to diagnostic criteria for depressive disorders. Participants rated how often they have been bothered by various symptoms associated with depression in the last 2 weeks on a 4-point Likert-type scale (0 = “Not at all” to 3 = “Every day”; total score 0–27). Scores over 10 indicate probable depression diagnosis. Internal consistency was acceptable (α = 0.76–0.88).

Post-traumatic stress disorder (PTSD) symptoms were measured with the PTSD Checklist for DSM-5 (PCL-5; Weathers et al., 2013), a 20-item self-report. Participants rated how much they have been bothered by symptoms associated with PTSD on a 4-point Likert-type scale (0 = “Not at all” to 3 = “Severely”). Total scores range from 0 to 60, and a cutoff of 33 was used to identify those with a probable PTSD diagnosis. Internal consistency was excellent (α = 0.93–0.94).

#### Treatment

Participants randomized to the treatment condition received up to 12 months of IMH-HV services. In this study, 33 (86.84%) of families who were assigned to the treatment condition received at least one IMH-HV session. The number of home visit sessions for those who received treatment across the 1-year intervention study ranged from one to 46 (*M* = 25.76; *SD* = 13.88). Visits were generally scheduled weekly, and visits lasted between 30 and 120 min on average (*M* = 70.93 min; *SD* = 17.87 min). Each visit included some of the following topics, depending on the current needs and readiness of the family: (1) Relationship building, (2) Providing for material needs, (3) Emotional support, (4) Developmental guidance, (5) Infant parent psychotherapy, (6) Helping clients to develop coping skills and social support.

Across the 12-month study, reasons for IMH-HV services ending were most commonly because treatment goals were met (45.46%), or because home visiting no longer worked with the family schedule (21.21%). If not already terminated by family request, treatment ended at the end of the study; however, some families indicated they would have continued with IMH-HV services if the trial was not ending.

Clinicians delivering IMH-HV services participated in a Learning Collaborative consisting of a 3-day training in the model, followed by biweekly coaching calls and follow up learning sessions to strengthen their understanding and application of the model. The training was provided by experts in the IMH-HV field. Throughout the study, clinicians also received weekly reflective supervision by a licensed clinical social worker who holds IMH-HV endorsement, and fidelity to the intervention was monitored using fidelity tracking forms; fidelity to the model was high (Huth-Bocks, et al., 2020).

In this study, 5 participants assigned to the treatment condition received no IMH-HV sessions; these participants were removed from the analysis for per-protocol analyses of treatment effect. Because this was a small sample and five participants represent 13% of the originally assigned treatment group, we believe that an intent-to-treat analysis would underestimate the effects of the treatment. Control group participants did not receive intervention, and self-referral to other interventions was monitored (of note, no family allocated to the control condition became connected to community-based IMH-HV services during the study).

#### Covariates

##### Current mental health treatment

At each time point, participants were asked if they were currently receiving mental health treatment (0 = no, 1 = yes).

##### Contextual stress

For analyses predicting change in mental health symptoms, a cumulative score representing an individual’s baseline level of contextual stress was used as a covariate. This index is based on work by Sameroff et al. (1987) and Sameroff et al. (1993) who showed that cumulative risk scores were very good predictors of later cognitive and socioemotional outcomes in children. Contextual stress was calculated by assigning a point for each of the following stress factors reported at baseline (prior to treatment): self-reported parenting stress at or above the 80th percentile; PTSD symptoms suggestive of possible PTSD diagnosis, the presence of significant childhood stress/trauma, and scores on a child abuse potential screening assessment suggestive of elevated risk of engaging in child abuse. The contextual stress score was moderately related to 12-month mental health outcomes (correlations with 12-month measures: PHQ: *r* = 0.34, *p* = 0.0046; GAD: *r* = 0.29, *p* = 0.019; PCL: *r* = 0.38, *p* = 0.0018).

##### Birth complications

Mothers were asked “Were there any complications at the target child’s birth?.” This was recorded as a binary response.

### Data analysis

For continuous measures of mental health symptoms, we used latent growth models, with treatment category as a predictor of the intercept and slope. We implemented the models in Mplus, Version 8.8, accounting for clustering by clinician, using TYPE = COMPLEX with robust maximum likelihood as an estimator. Baseline contextual stress and the binary measure of birth complications were included as intercept and slope predictors; current mental health treatment was tested as a covariate for the mental health outcome at each time point and retained if it was related at *p* < 0.10. We explored using all five time points (baseline, 3, 6, 9 and 12 months) and, to simplify the modeling due to our small sample size, using three time points (baseline, 6, and 12 months).

Because recent guidelines advise against describing research results using a “bright line” cut off of *p* = 0.05 and the words “significant” and “non-significant,” we report all *p* values as continuous (e.g., *p* = 0.017 versus *p* < 0.05) (Amrhein et al., 2019; Wasserstein et al., 2019) and we report all the results of analyses whether or not *p* < 0.05.

## Results

**Figure 1:**
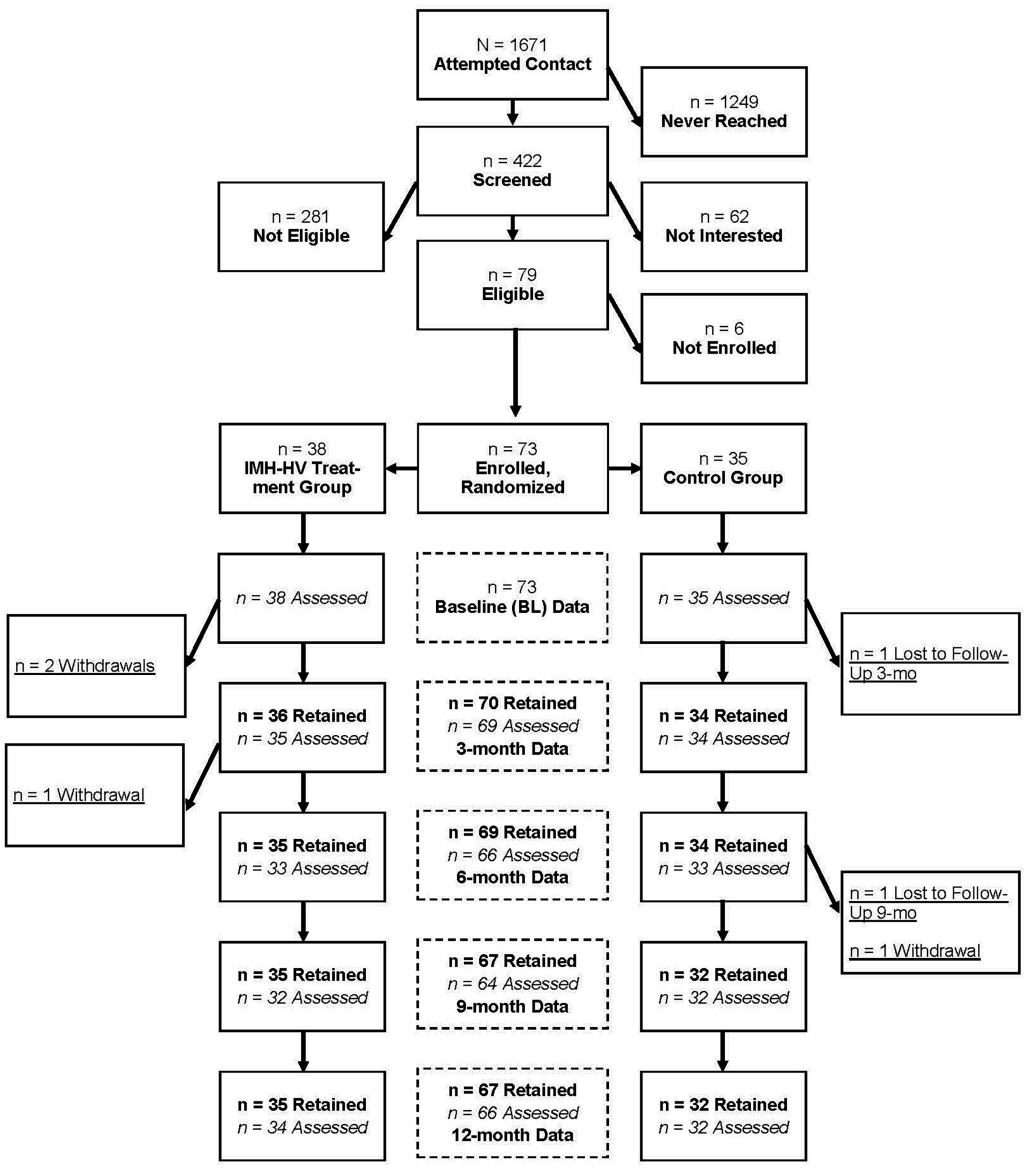
Participant flow diagram.

The participant flow diagram is shown in Figure 1. Retention was excellent, with 90% of the randomized sample being retained at 12 months. Retention was very similar in treatment (89%) and control groups (91%). In addition, demographic characteristics including marginalized identity and income did not predict retention (logistic regression, *p* = 0.73, *p* = 0.60, respectively). Similarly, baseline levels of depression, anxiety, and adverse childhood experiences did not predict retention (logistic regression, *p* = 0.74, *p* = 0.87, *p* = 0.46, respectively). Sample demographic information can be found in Table 2 for treatment and control samples. Maternal age (*M* = 31.91; *SD* = 10.76) ranged from 19 to 44 years; child age (*M* = 10.76; *SD* = 7.41) ranged from 0 to 24 months at study entry. Most of the participants were married (69.86%). Annual family income ranged from less then $5000 to over $100,000, but economic need was relatively high, with approximately half of the sample being eligible for and receiving public insurance and WIC and approximately one-third of the sample receiving additional food assistance. Children in the sample were predominantly White (72.60%) or Black (36.99%); participants could identify multiple race/ethnicity descriptors. When compared to the expected general population of those receiving IMH-HV in the community, the current sample is somewhat older with an average age 31.4 (compared to 27.1 in a community sample; Jester et al., 2023). The current sample was much more likely to be married (68% vs. 22% in the community sample) and the income profile was higher (in the community sample, 68.3% had income under $20,000 vs. 27% in this sample). This is due to the fact that in the community-served population, all families must be eligible for Medicaid to receive services. Although the current sample has less demographic risk, the mothers are similar with respect to mental health symptoms at baseline. In the current sample, the mean number of PHQ symptoms is 9.45 and in the community sample it was 9.83; PCL symptoms for the current sample were 22.89, compared to community sample of 24.83 (anxiety symptoms were not measured in the community sample).

**Table 2:** Sample demographics.

Characteristic	Total sample (73)		Treatment sample (33)		No treatment (40)		*p-*value difference
	Range	*M* (*SD*)	Range	*M* (*SD*)	Range	*M* (*SD*)	
Maternal Age (years)	19–44	31.91 (5.69)	21–42	31.75 (5.74)	19–44	32.05 (5.71)	0.81
Child Age (months)	0–24	10.76 (7.41)	0–24	10.14 (7.17)	0–24	11.28 (7.66)	0.65
	*n* (%)		% (*n*)		% (*n*)		
Child gender	Male – 43.94% Female – 56.06%		Male – 58.62% Female – 41.38%		Male – 32.43% Female – 62.50%		0.03
Family composition							
Married	51 (69.86%)		23 (69.70%)		28 (70.00%)		0.98
Divorced	7 (9.59%)		3 (9.09%)		4 (10.00%)		0.87
Separated	3 (4.11%)		0 (0.00%)		3 (7.50%)		0.11
Single (Never married)	20 (27.40%)		10 (30.30%)		10 (25.00%)		0.61
Family income variables							
Household Income <$20,000	20 (27.39%)		10 (30.30%)		10 (25.00%)		0.64
Currently receive medicaid	33 (45.21%)		14 (42.42%)		19 (47.50%)		0.65
Currently receive food assistance	24 (32.88%)		11 (33.33%)		13 (32.50%)		0.94
Currently receive WIC	38 (52.05%)		15 (45.46%)		23 (57.50%)		0.31
Child race/Ethnicity							
White	53 (72.60%)		25 (75.76%)		28 (70.00%)		0.44
Black	27 (36.99%)		11 (33.33%)		16 (40.00%)		0.62
Hispanic or Latino/a	9 (12.33%)		6 (18.19%)		3 (7.50%)		0.15
Other/Not Specified	13 (17.81%)		6 (18.19%)		7 (17.50%)		0.98

Participants could choose as many race/ethnicity indicators as needed to describe their child’s race/ethnicity and as many relationship status options as applied; totals are not meant to equal 100%.

**Figure 2:**
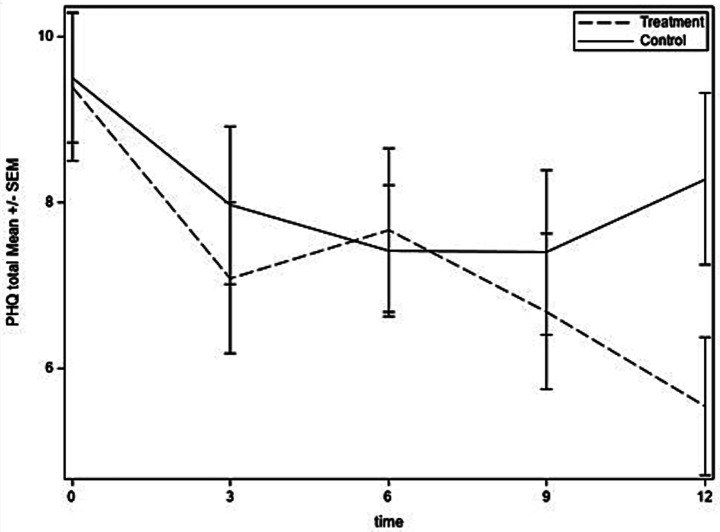
Depression symptoms over time for treatment and control groups.

**Figure 3:**
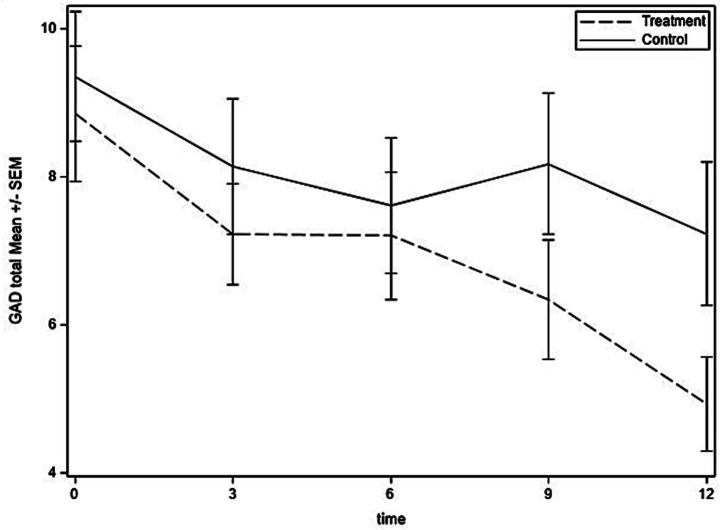
Anxiety symptoms over time for treatment and control groups.

**Figure 4:**
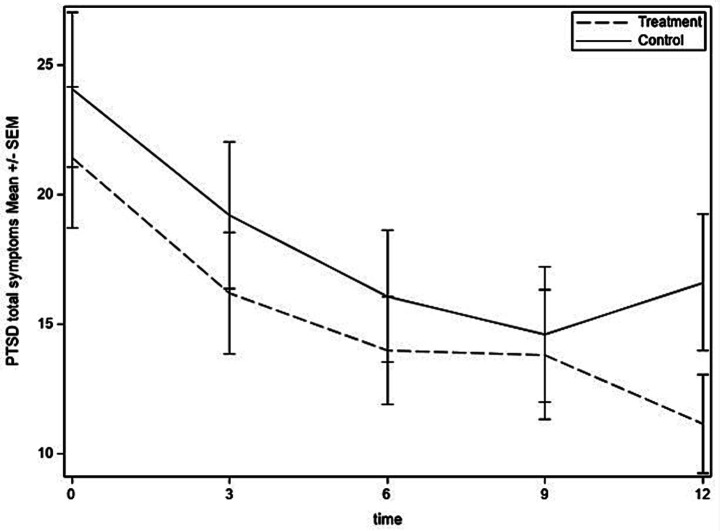
PTSD symptoms over time for treatment and control groups.

Figures 2–4 show the time courses of depression, anxiety, and PTSD symptoms, respectively, in the treatment and control groups. Means are based on those remaining in the study at each time point [baseline: 73; 3 months: 69 (95%); 6 months: 66 (90%); 9 months: 64 (88%); and 12 months: 66 (90%)]. Depression symptoms decreased for both groups from baseline to 6 months but then leveled off in the control group and continued to decrease to the 12-month time point for the treatment group. For anxiety, treatment and control groups followed similar trajectories through 6 months, at which time the treatment group continued to decrease in anxiety symptoms, whereas the control group had a slight uptick in symptoms at 9 months and then decreased at 12 months. PTSD symptoms had similar trajectories in treatment and control groups until 9 months, then the treatment group experienced some improvement whereas the control group experienced worsening of symptoms.

For latent growth modeling, we tested linear models with three time points (baseline, 6- and 12-month follow up) and with five time points (adding 3-month and 9-month assessments). For each model, the AIC and BIC were lower, indicating better fit, for the 3-time point models. Specifically, for the PHQ-9, a three time point model had AIC = 1116.70; the model with five time points had AIC = 1806.60. For the GAD-7, the three time point model had an AIC = 1134.00; the five time point model had an, AIC = 1820.97. The PCL three time point model had an AIC = 1519.76; the five time point model had an, AIC = 2456.14. Parameters for the effect of being in the per-protocol treatment group are shown in Table 3. None of the models showed large effects of treatment group on intercept (standardized coefficients ranged from 0.029 to 0.08, with *p* > 0.65 for all) whereas the coefficients for slope were more substantial, ranging from –0.46 to –0.57, with *p-*values ranging from 0.015 to 0.085. These results provide some evidence of a treatment effect, since the mental health indicators start at similar levels (no difference in intercepts between groups) and decrease more rapidly in the group that received treatment (differences in slopes, predicted by treatment group). In intent-to-treat analysis (ITT), we saw similar but weaker effects on slope. The standardized coefficients for slope were: for depression: −0.38 (*p* = 0.079), for anxiety: −0.27, *p* = 0.36; for trauma symptoms: −0.49, *p* = 0.044. The ITT models showed similar lack of effects for intercept: depression (0.043, *p* = 0.84), anxiety (0.29, *p* = 0.32), trauma symptoms (2.23, *p* = 0.23).

**Table 3:** Parameters from Latent Growth Models (Coefficient for per-protocol treatment group).

Outcome	Intercept raw coefficient	Intercept standardized coefficient	*p*-value	Slope raw coefficient	Slope standardized coefficient	*p*-value
Depression	0.33	0.08	0.69	−0.19	−0.57	0.015
Anxiety	0.10	0.029	0.90	−0.13	−0.53	0.085
PTSD	0.74	0.074	0.78	−0.43	−0.46	0.057

## Discussion

Our primary goal was to evaluate the impact of a relational, home-based intervention program on maternal mental health symptoms. Specifically, we examined how receipt of the Michigan Model of Infant Mental Health Home Visiting (IMH-HV) was associated with change in maternal symptoms of depression, anxiety, and trauma. We found that mental health symptoms decreased for mothers who received IMH-HV services compared to those who did not receive intervention. The impact on depression symptoms is key, as federal initiatives, including the Maternal, Infant, and Early Childhood Home Visiting (MIECHV) Program focus on improving maternal and child health. Home visiting models, although varied in strategy, often share common goals to intervene early, engage with parents in their parenting roles, strengthen protective factors, and reduce risk factors, which often include improving maternal mental health to promote healthy parent–child relationships and children’s development (Ammerman et al., 2013; Minkovitz et al., 2016).

Previous research has demonstrated mixed findings regarding the impact of home-based interventions on maternal depression, with some interventions demonstrating a reduction in depressive symptoms either directly (McFarlane et al., 2017; Tandon et al., 2020)or indirectly (Chazan-Cohen et al., 2007; Sandner et al., 2018). Less literature identifies home visiting programs’ effectiveness in reducing maternal anxiety or trauma symptoms, with a few exceptions (e.g., Lavi et al., 2015). As such, our demonstration that IMH-HV intervention leads to reductions of depression, anxiety, and trauma symptoms is an important contribution to the literature and suggests that relationally-based interventions delivered in the home that have positive impacts on parenting, parent–child relationships, and child development can also improve maternal mental health.

There are many components to IMH-HV treatment, which are provided flexibly and in response to family needs. Subsequently, there are many possible mechanisms by which this intervention model demonstrated positive effects on maternal mental health. A core component of IMH-HV is infant-parent psychotherapy, which is an intervention component that attends to the parent–child relationship and has been linked with increased parenting sensitivity and parent awareness of their own and their babies’ mental states. Previous evaluations of home-based interventions utilizing parent-infant psychotherapy approaches have demonstrated reductions in maternal depression (Fonagy et al., 2016; Huang et al., 2020). Results of this study expand upon those findings but also identify reduction in anxiety symptoms resulting from the intervention. Other aspects of IMH-HV intervention include developmental guidance, and attention to child development, which other programs have found to mediate the effect of home-based intervention on improved maternal depression (Chazan-Cohen et al., 2007). As a treatment model that attends to multiple generations, IMH-HV may capitalize on the bidirectional, compounding effects of the intervention, which may result in improved maternal mental health. Studies with larger sample sizes will help to identify aspects of IMH-HV that drive improvements in maternal mental health.

This study is not without its limitations. As is the case with many clinical trials, the sample size was constrained, despite excellent retention of participants. As such, this study evaluated the effect of intervention received rather than evaluating differences between treatment conditions as assigned. Additionally, although IMH-HV services in communities are delivered to all caregivers, including fathers and foster/adoptive parents, this sample is comprised exclusively of biological mothers, limiting generalizability. It may be that IMH-HV has different effects on other caregiver’s mental health symptoms. This study examines the impact of the intervention on mental health symptoms immediately post-intervention; future studies should expand follow-up assessments to identify how reductions in mental health symptoms following IMH-HV treatment are sustained over time. Finally, and directly related to the findings described in this paper, in community settings, IMH-HV services may be delivered until the child reaches 3 years. Within this study, treatment was constrained to 12 months during the study period. It is possible that in community settings where treatment can continue for longer than 12 months, the trend of decreasing anxiety and trauma symptoms might result in stronger reductions for anxiety and trauma at the end of treatment. Future studies should examine this.

## Data Availability

The raw data supporting the conclusions of this article will be made available by the authors, without undue reservation.

## Group members of Michigan Collaborative for Infant Mental Health Research

The Michigan Collaborative for Infant Mental Health Research (MCIMHR) is composed of researchers from eight universities and from the Alliance for the Advancement of Infant Mental Health, whose efforts are aligned with the promotion of research of Infant Mental Health. Core MCIMHR members have collaborated in the design, implementation and/or dissemination of the current study. MCIMHR core members include (in alphabetical order): Emily Alfafara, Carla Barron, Holly E. Brophy-Herb, Nora L. Erickson, Hiram E. Fitzgerald, Alissa C. Huth-Bocks, Meriam Issa, Jennifer M. Jester, Megan M. Julian, Jamie M. Lawler, Rena Menke, Alyssa S. Meuwissen, Alison L. Miller, Maria Muzik, Larissa N. Niec, Jerrica Pitzen, Julie Ribaudo, Jessica Riggs, Katherine L. Rosenblum, Sarah E. Shea, Paul Spicer, Ann M. Stacks, Chioma Torres, Laurie Van Egeren, Rachel Waddell, Christopher L. Watson, Deborah J. Weatherston and Kristyn Wong.
